# Radiomodulatory and antioxidant properties of a novel pyrazine-linked quinazolinone sulfonamide derivative: Insights into renal apoptosis

**DOI:** 10.1007/s00210-026-05248-2

**Published:** 2026-04-09

**Authors:** Heba M. Karam, Mai H. Mekkawy, Amira Abd-ElRaouf, Aiten M. Soliman, Mostafa M. Ghorab

**Affiliations:** Drug Radiation Research Department, National Center for Radiation Research and Technology (NCRRT), Egyptian Atomic Energy Authority, Cairo, 11787 Egypt

**Keywords:** Antioxidant, Scavenging, Kidney damage, Apoptosis, Gamma radiation, Quinazolinone

## Abstract

Quinazolinone derivatives possess a privileged scaffold with variable pharmacological activities. This study conducted an *in vitro* evaluation of the pyrazine-linked quinazolinone sulfonamide derivative (**PQS**) to assess its anticancer activity, following confirmation of its safety in a normal cell line. Results revealed that **PQS** has a noteworthy cytotoxic effect on HepG-2 and HeLa cells, indicating its potential anticancer activity. Additionally, **PQS** is quite safe on normal Vero cells. Furthermore, *in vivo* biological activity of **PQS** was evaluated for its radioprotective properties in (5 Gy) irradiated mice. **PQS** showed mitigation of gamma radiation-induced oxidative stress, verified by the decline in MDA, ROS and NO levels, while GSH levels and GST activities were improved in kidney tissues. The apoptotic pathway was also assessed, where weak expression of caspase-3 and Bcl2-associated X protein (Bax) was noticed paired with higher expression of B-cell lymphoma 2 (Bcl2) after **PQS** treatment in irradiated mice. These results were confirmed by histopathological examination of kidney tissues. Besides, *in-silico* ADME studies revealed that compound **PQS** showed drug-like properties, confirmed by its physicochemical and pharmacokinetic properties. In conclusion, the results of the present study demonstrated that **PQS** has dual activity as a potential anticancer on HepG-2 and HeLa cancer cell lines and radio-protective activity through its anti-apoptotic and antioxidant action in the *in vivo *study.

## Introduction

To date, significant adverse effects experienced during chemotherapy and/or radiotherapy are considered of interest in cancer research (Chandra et al. 2021; Ingrand et al. 2020; Yazbeck et al. 2022). Conventional anticancer drugs, while effective against tumor cells, often exert substantial toxic effects on normal cells. The cytotoxic side effects of traditional anticancer drugs arise primarily from their lack of specificity for cancer cells. This harms the normal tissues and subsequently causes a range of side effects that negatively impact patients' quality of life and can limit the effectiveness of cancer treatment (Ingrand, et al. 2020). Significant side effects related to damage of normal cells might include: cardiotoxicity (Abd-ElRaouf et al. 2021; Herrmann 2020), nephrotoxicity (Małyszko et al. 2017), neurotoxicity (Was et al. 2022) and gastrointestinal toxicity (Remesh 2012). Radiation therapy, like many chemotherapeutic agents, increases oxidative stress and can harm healthy tissues, generating large quantities of reactive oxygen species (ROS) that can injure DNA and cell membranes in both cancerous and normal tissues (Azzam et al. 2012; Cauli 2021; Jiang et al. 2023; Kasumbwe and Mohanlall 2025; Zhao and Robbins 2009). Thus, there is an urgent need for intensive research to offer safer anticancer agents with intrinsic antioxidant activity or adjunct therapies that better protect normal cells and ultimately improve quality of life and treatment outcomes for patients.

Antioxidants can scavenge excess ROS, reducing DNA, protein and lipid damage in normal cells. At the same time, reducing adverse effects allows for higher or prolonged dosing and increases the effectiveness of cancer treatments. To be effective against cancer, anticancer drugs are provided in high doses, but are limited by side effects. Adjuvant therapy could promise to allow for safer dosing, thus antioxidants could counteract this effect and enhance the therapeutic window (Mathijssen et al. 2014; Papachristos et al. 2023).

Importantly, dual or context-dependent activity of bioactive compounds has been widely documented, where compounds induce cytotoxic action in cancer cells while protecting normal tissues from oxidative or radiation-induced damage. This selectivity is attributed to differences in redox balance, mitochondrial susceptibility, and apoptotic signaling thresholds between malignant and normal cells (Snezhkina et al. 2019; Trachootham et al. 2009). Cancer cells often exhibit higher basal oxidative stress and altered mitochondrial function, making them more susceptible to apoptosis induction, whereas normal cells may benefit from antioxidant and anti-apoptotic protection.

Some naturally derived compounds, such as curcumin, resveratrol, and certain flavonoids, exhibit both cancer-inhibitory and antioxidant properties, making them attractive as safer alternatives or adjuncts to established regimens. Most of these natural products have low bioavailability, either due to low absorption (eg: curcumin) or rapid liver metabolism (eg: resveratrol), furthermore, they exert weak anticancer properties, hence direct the search for developing new agents through a slight alteration in their structure to modify anticancer drugs with a different moiety to be effective as an antioxidant while preserving their anticancer properties (Kapadia et al. 2002; Yang et al. 2001).

**Fig. 1:**
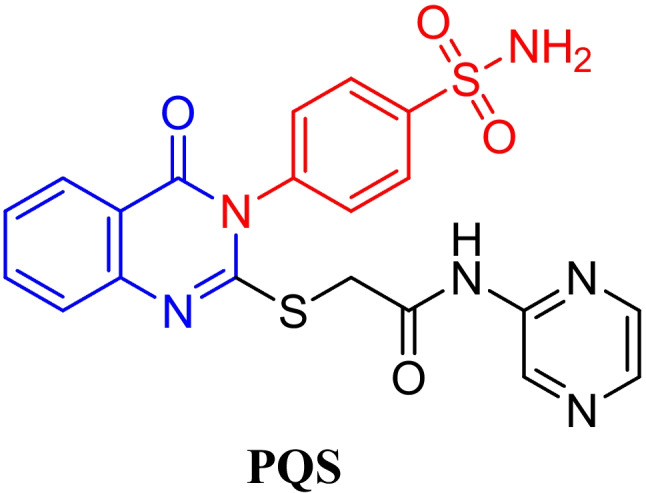
The chemical structure of **PQS**

Quinazolinone and sulfonamide derivatives are interesting motifs that possess a privileged scaffold exhibiting variable pharmacological activities, including antioxidant (Soliman et al. 2025, 2023, 2020a), free radical scavenging abilities, as well as anticancer properties (Ghorab et al. 2018, 2021, 2023; Soliman et al. 2019b). In view of one of our previous studies that synthesized novel quinazolinone derivatives bearing a benzenesulfonamide moiety with variable heterocyclic tail and their structures were established on the basis of spectral data. The *N*-(pyrazin-2-yl)−2-[(4-oxo-3-(4-sulfamoylphenyl)−3,4-dihydroquinazolin-2-yl) thio] acetamide **(PQS)** was identified in a screen of 14 quinazolinone–sulfonamide derivatives as the most potent candidate, with the highest antioxidant activity (DPPH IC50 = 45.76 µM). Acute toxicity studies indicated a relatively safe profile (LD50 = 200 mg/kg). Characterization of **PQS** was done using TLC,,melting point, ^1^H NMR, , ^13^C NMR, FT-IR and elemental analysis. In irradiated mice, **PQS** alleviated oxidative stress by reducing MDA, ROS and NF-κB levels while enhancing SOD and PON1 activities and restoring hepatic Zn^2^⁺ levels. As well, docking studies revealed favourable binding with SOD and PON1 active sites. These effects may relate to the known antioxidant mechanisms of sulfonamide, pyrazine and quinazolinone scaffolds, known for their free radical scavenging, metal chelation (Fig. 1) (Soliman et al. 2020a).

Therefore, this study aimed to explore the potential anticancer activity of **PQS** on cancer HepG-2 and HeLa cell lines as well as examine the safety of **PQS** on normal Vero cells, on the way to *in vivo* investigation of its ability to ameliorate radiation-induced kidney apoptosis and its radio-modulating potential activity in irradiated mice.

This was carried out through the estimation of the serum urea and creatinine levels, as well as the determination of kidney malondialdehyde (MDA), ROS and nitric oxide (NO) levels, along with reduced glutathione (GSH) level and the activity of Glutathione-S-transferase (GST). Likewise, apoptosis pathway was assessed, where expression of caspase-3, Bax and Bcl2 was determined after **PQS** treatment of irradiated mice. That was confirmed by histopathological examination. As well as, *in-silico* pharmacokinetic properties of **PQS** were determined using an ADME study.

## Materials & methods

### In vitro* biological evaluation*

#### Cell lines

The Vero cells isolated from African green monkey kidney epithelial cell (Cat# CCL-81), Human Hepatocellular Carcinoma Cell Line (HepG-2) isolated from human hepatocellular carcinoma epithelial cell (Cat#HB-8065) and Henrietta Lacks Carcinoma Cell Line (HeLa) isolated from human adenocarcinoma epithelial cell (Cat#CCL-2) were used. Cell lines were purchased from the Tissue Culture Unit of The Holding Company for Biological Products and Vaccines (VACSERA), Cairo, Egypt. The cells for this investigation were obtained from the American Type Culture Collection (ATCC). They were cultured in RPMI-1640 medium supplemented with 10% fetal bovine serum (FBS) at 37 °C in a humidified atmosphere containing 5% CO_2_ and 95% air. All the biosafety instructions & precautions were implemented and strictly followed throughout the experimental work procedures. At the end of the experiment, all cultures, isolates and remains of potentially biologically hazardous materials were safely disposed of via autoclaving.

#### MTT cytotoxicity assay

The effect of **PQS** on viability of normal Vero cells, as well as HeLa & HepG-2 cancer cell lines, was evaluated using the MTT cytotoxicity assay (Alley et al. 1988; Van de Loosdrecht et al. 1994). The viability of Vero normal cells was compared to ascorbic acid as a standard antioxidant compound, while HeLa & HepG-2 cancer cells were compared to doxorubicin as a standard anticancer drug. The 96-well tissue culture plate was inoculated with 1 × 10^5^ cells/mL (100 µL/well) and incubated at 37 °C for 24 h. Samples of **PQS** or ascorbic acid, ranging in concentration from 1.25 to 0.156 µM, were employed on Vero cell line. While in HepG-2 and HeLa cell lines, **PQS** and doxorubicin were supplemented in a concentration range from 100 to 3.125 µg/ml. MTT solution was prepared (5 mg/mL in PBS) (BIO BASIC CANADA INC). The data were presented as mean ± SD (n = 3), and the half maximum inhibitory concentrations (IC_50_) were computed.

### In vivo* biological evaluation*

#### Animals

Thirty-two male albino BALB/c mice (25–30 ± 2.5 g) were obtained from the animal house of the National Center for Radiation Research and Technology (NCRRT), Cairo, Egypt. Animals were left one week to acclimatize in lab environment before starting the onset of the experiment. They were kept under controlled environmental conditions; room temperature (24–26 ºC), constant humidity (60 ± 10%), with alternating 12 h dark and light cycle. Water and food were allowed ad libitum. Mice were treated, held and administered the drug by trained staff, efforts were made to minimize their suffering.

All animal procedures were performed in accordance with the Ethics Committee for Animal Experimentations of the NCRRT (Permit Number: 61A/23), which complies with the Guide for Care and Use of Laboratory Animals issued by the CIOMS and ICLAS International Guiding Principle for Biomedical Research Involving Animals 2012.

#### Irradiation process

Mice received a single dose of 5 Gy whole-body gamma irradiation. The dose selected was according to (Alkis et al. 2021). The dose rate was 0.354 Gy/min at the time of exposure. The irradiation process was carried out at NCRRT using a Gamma cell-40 biological radiator with a ^137^Cs source (Atomic Energy of Canada Limited; Sheridan Science and Technology Park, Mississauga, Ontario, Canada). The irradiation chamber’s dimensions are 10 cm (height) X 40 cm (diameter), which accommodates a complete animal group at a time. It is characterized by a uniform distribution of rays. The Dosimetry Department members at the NCRRT have carried out the dose validation on a scheduled basis to confirm the dose rate of the gamma-ray source, the absorbed dose received by mice and the uniformity of dose were done via dose mapping measurements.

#### Experimental design

Thirty-two mice were randomly classified into four groups (n = 8/group). Group I (Control): mice were injected with 10% DMSO i.p for 3 days (Modrzyński et al. 2019). Group II (**PQS**): mice were injected with (20 mg/kg, i.p:1/10 LD_50_) of compound **PQS** for three consecutive days (Soliman et al. 2020a). Group III (IRR): animals exposed to 5 Gy whole-body gamma irradiation and were injected with 10% DMSO i.p for 3 days (Alkis et al. 2021). Group IV (**PQS** + IRR): the first day, mice received **PQS** (20 mg/kg, i.p.) and after 2 h, were exposed to 5 Gy, then received **PQS** for the next 2 consecutive days. After 24 h of the last injection, mice were deeply anesthetized using urethane (1.2 mg/kg i.p) (Flecknell 1993). Blood samples were collected by heart puncture and then euthanized by cervical dislocation. Blood from each animal was separated into two sterile test tubes: one containing ethylenediamine tetraacetic acid (EDTA) as an anticoagulant for flow cytometry, and the second was plain, to be centrifuged at 3000 rpm for 15 min. Then, serum was isolated and kept at −80 °C for further biochemical tests. Two kidneys were separated out, washed with ice-cold deionized water, dried on filter paper and weighed. One kidney was homogenized in ice-cold 0.1 M phosphate buffer saline (pH 7.4) and stored at −80 ˚C until used for subsequent biochemical analysis and the other was fixed in 10% buffered formalin for histopathological and immunohistochemical examinations.

#### Biochemical parameters investigated in serum

Serum urea and creatinine levels were estimated for assessment of renal function using a colorimetric cell-based ELISA kit (UR 2110 & CR1250, respectively, Biodiagnostic Company, Dokki, Giza, Egypt), following the recommendations of the International Federation of Clinical Chemistry.

#### Biochemical parameters investigated in kidney homogenate

Lipid peroxidation level in the kidney was assessed by measuring MDA as an indicator using the method of Yoshioka et al. (Yoshioka et al. 1979). The generation of ROS in kidney tissues was measured according to the method of Vrablic et al. (Vrablic et al. 2001). Kidney homogenates were also used for measuring NO content by the method of Miranda et al*.* (Miranda et al. 2001). Estimation of the total GSH was according to the Beutler method (Beutler et al. 1963). Activity of GST was measured using the method of Mannervik et al. (Mannervik et al. 1985).

#### Flow cytometry-based apoptosis detection

The apoptotic detection of peripheral blood mononuclear cells was carried out according to the method of Cui et al. (Cui et al. 2021). Blood samples were stained with Annexin V‐FITC/PI using Apoptosis Detection Kit (Cat#556547; BD Biosciences). Flow cytometry was performed to quantify cell types using fluorescence‐activated cell sorting (FACS) buffer on an LSRII instrument (BD Biosciences). Data analysis was done using the Flow Jo program (Biosciences 2011).

#### Histopathological examination of Kidney

Kidneys were removed directly after scarification, weighed and one of them was fixed in a 10% formalin solution. After 24 h, the tissue was embedded in a paraffin block after dehydrating with increasing concentrations of 70–100% ethanol and clearing in xylene. Then sectioned at 4–6 μm thick. The obtained tissue sections were de-paraffinized using xylol and placed on a glass slide, stained using Hematoxylin and Eosin. Stained tissue sections were observed through the electric light microscope (Bancroft and Gamble 2008).

#### Immunohistochemistry analysis

Histopathological processing and assessment of specimens were performed with the aid of a skilled histologist, blinded to the studied sample’s identity to avoid any bias. The immunohistochemistry data of caspase-3, Bax & Bcl2 were scored in the kidney tissues in different areas, according to (Bebars et al. 2017). Reactivity was mainly the number of cytoplasmic nuclear staining per high power field of the specimens.

Paraffin-embedded, 5 μm thick sections of kidney from different animal groups were rehydrated, then treated for 20 min with 3% H_2_O_2_. Sections were pre-treated in citrate buffer (pH 6.0) in a microwave. Then, sections were incubated overnight at room temperature with caspase-3 polyclonal antibodies at 1:200 dilution (Cat# 25128–1- AP Proteintech), Bax polyclonal antibodies at a dilution of 1:2000 (Cat#50599–2-Ig Proteintech) and Bcl2 polyclonal antibodies at 1:200 dilution (Cat #26593–1-AP Proteintech) for an hour at room temperature. Subsequently, sections were rinsed and incubated with a secondary antibody prior to visualization with diaminobenzidine (DAB) as substrate, then washed, and slides were counterstained with hematoxylin. Negative control slides were obtained by skipping the primary antibody incubation step. Afterward, slides were visualized under a light microscope and the extent of cell immunopositivity was assessed. Using image analysis software, the positive brown region of each marker's expression was quantified as an optical density in 7 high-power microscopic fields (Image J, 1.46a, NIH, USA).

### Physico-chemical and pharmacokinetic parameters (*In-silico* ADME study)

All physicochemical parameters and pharmacokinetics were calculated *in silico* using SwissADME software (Daina et al. 2017).

### Statistical analysis

Data were expressed as mean ± S.D. Comparisons between means were carried out using one-way ANOVA followed by Bonferroni post hoc comparisons test at p < 0.05. Statistical analysis was performed using GraphPad Prism software package version 5 (GraphPad Software Inc., USA).

## Results

### *In vitro evaluation (MTT cytotoxicity assay)*

**Fig. 2:**
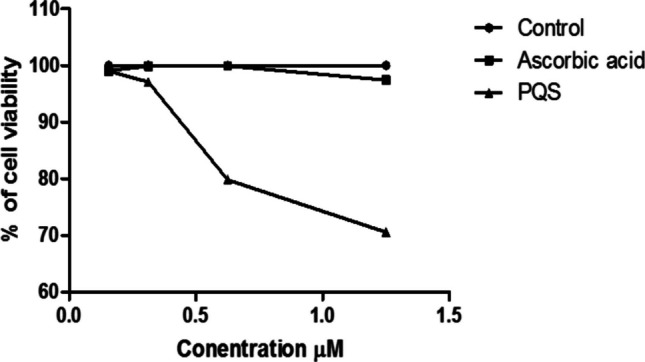
Effect of **PQS** and Ascorbic acid on cell viability of Vero normal cell lines. Data were expressed as mean ± S.D. (n = 3). Viability: surviving normal Vero cells

**Fig. 3:**
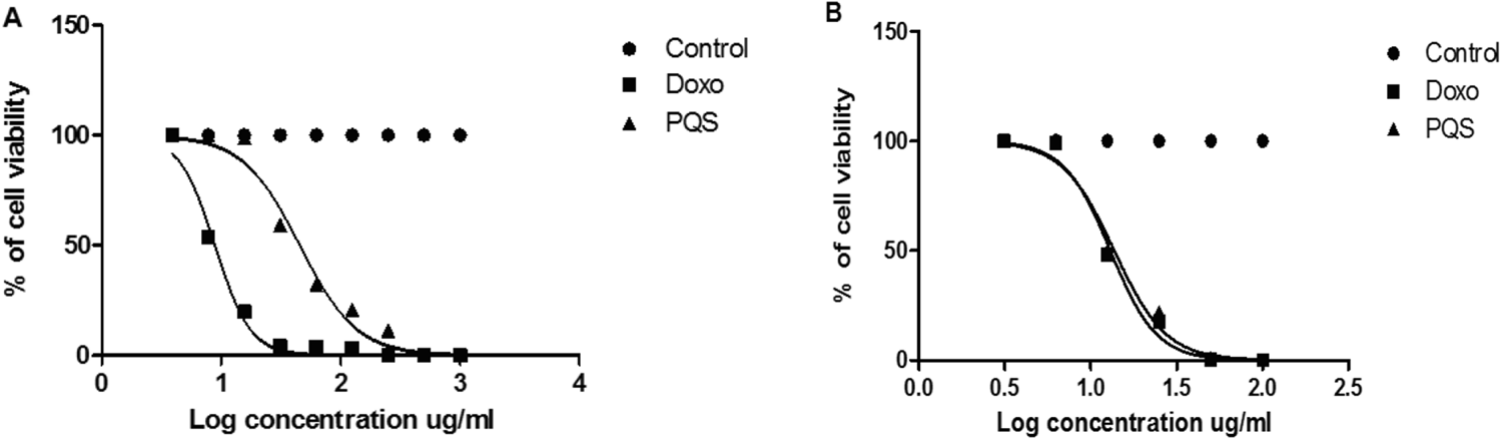
Effect of **PQS** and doxorubicin on cell viability of (**A**) HepG-2 and (**B**) HeLa cell lines. Data were expressed as mean ± S.D. (n = 3). Viability: surviving cancer cells

The viability of Vero cell line against **PQS** confirmed its safety on normal cells, the viability differences between PQS and ascorbic acid at each concentration were statistically non-significant, which would clarify the safety of the compound (Fig. 2). Hence, **PQS** maintains viability from 70.57 to 99% at the tested concentrations, supporting its selection for further *in vivo* biological evaluation, aiming to estimate its ability to ameliorate radiation-induced kidney damage. Moreover, the viability of HepG-2 cancer cell line was assessed against **PQS**, IC_50_ was 14.12 ± 0.05 μM in comparison to the standard drug, doxorubicin (IC_50_ 1.98 ± 0.01 μM). In addition, IC_50_ of **PQS** on HeLa cell line was 5.06 ± 0.02 μM, compared to doxorubicin (IC_50_ 4.22 ± 0.02 μM) (Fig. 3).

### *In vivo evaluation*

Four groups of mice were used: Group I as control, Group II exposed to 5 Gy of gamma radiation, Group III received compound **PQS** for 3 consecutive days and Group IV received compound **PQS** and was exposed to 5 Gy.

#### Assessment of kidney function test

**Fig. 4:**
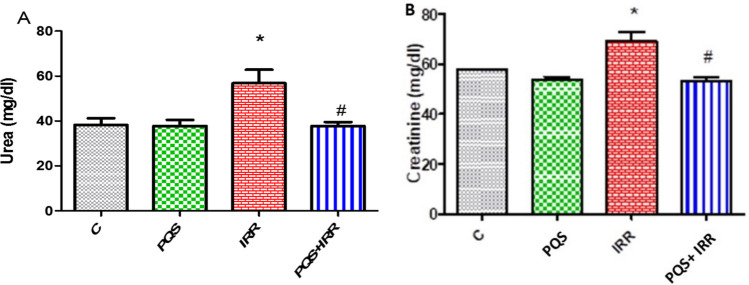
Effect of compound **PQS** on serum (**A**) Urea and **(B)** Creatinine in irradiated mice. The results were expressed as mean ± S.D. Statistical analysis was carried out by one-way ANOVA followed by a Bonferroni post hoc comparison test. *: significantly different from control group, #: significantly different from irradiated group at *p* < 0.05. (n = 8)

The irradiated animals showed a significant increase in urea and creatinine levels by (48.65% and 20.37%), respectively, as compared to normal mice. In the meantime, treatment of irradiated mice with **PQS** significantly reduced urea and creatinine levels by 33.46% and 23.76%, respectively, compared to the irradiated group (Fig. 4A **& B)**. Thus, **PQS** appears to protect renal function against radiation-induced damage, supporting its potential nephroprotective effect.

#### Assessment of oxidative status of kidney homogenate tissue

The measurements of MDA, ROS, NO content, GST activity and GSH content are important, as these play a vital role in the mechanisms of radiation toxicity. The results obviously showed that gamma radiation induced renal oxidative stress, confirmed by a significant increase in the levels of MDA (by 136.5%), ROS (by 37.4%), and NO content (by 94.7%) from control group. It was noticed that radiation group showed a decrease in GSH level (by 9.5%) as well as a decline in the activity of GST (by 63.2%) as compared to the normal group.

**Fig. 5:**
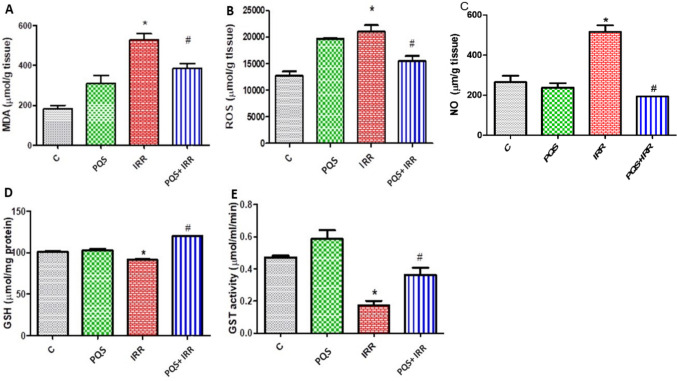
Effect of **PQS** on kidney homogenate of irradiated mice (**A**) lipid peroxidation measured as MDA, (**B**) ROS levels, (**C**) NO content, (**D**) GSH content and (**E)** the activity of GST. The results were expressed as mean ± S.D. Statistical analysis was carried out by one-way ANOVA followed by a Bonferroni post hoc comparison test. *: significantly different from control group, #: significantly different from irradiated group at *p* < 0.05. (n = 8)

In the meantime, compound **PQS** amended the oxidant status distressed by gamma radiation. Where, PQS + IRR group showed a significant decline in the levels of MDA (by 27.55%) and ROS (by 26.18%), as well as decline in NO content (by 152.60%) likewise upsurge in GST activity (by 109.1%) and rise in GSH content (by 31.3%), when compared to the irradiated group (Fig. 5** A, B, C, D & E**). Thus, PQS’s antioxidant effect may contribute to renal protection against radiation-induced oxidative damage (Fig. 5**)**.

#### Detection of apoptosis in peripheral blood mononuclear cells (PBMCs)

**Fig. 6:**
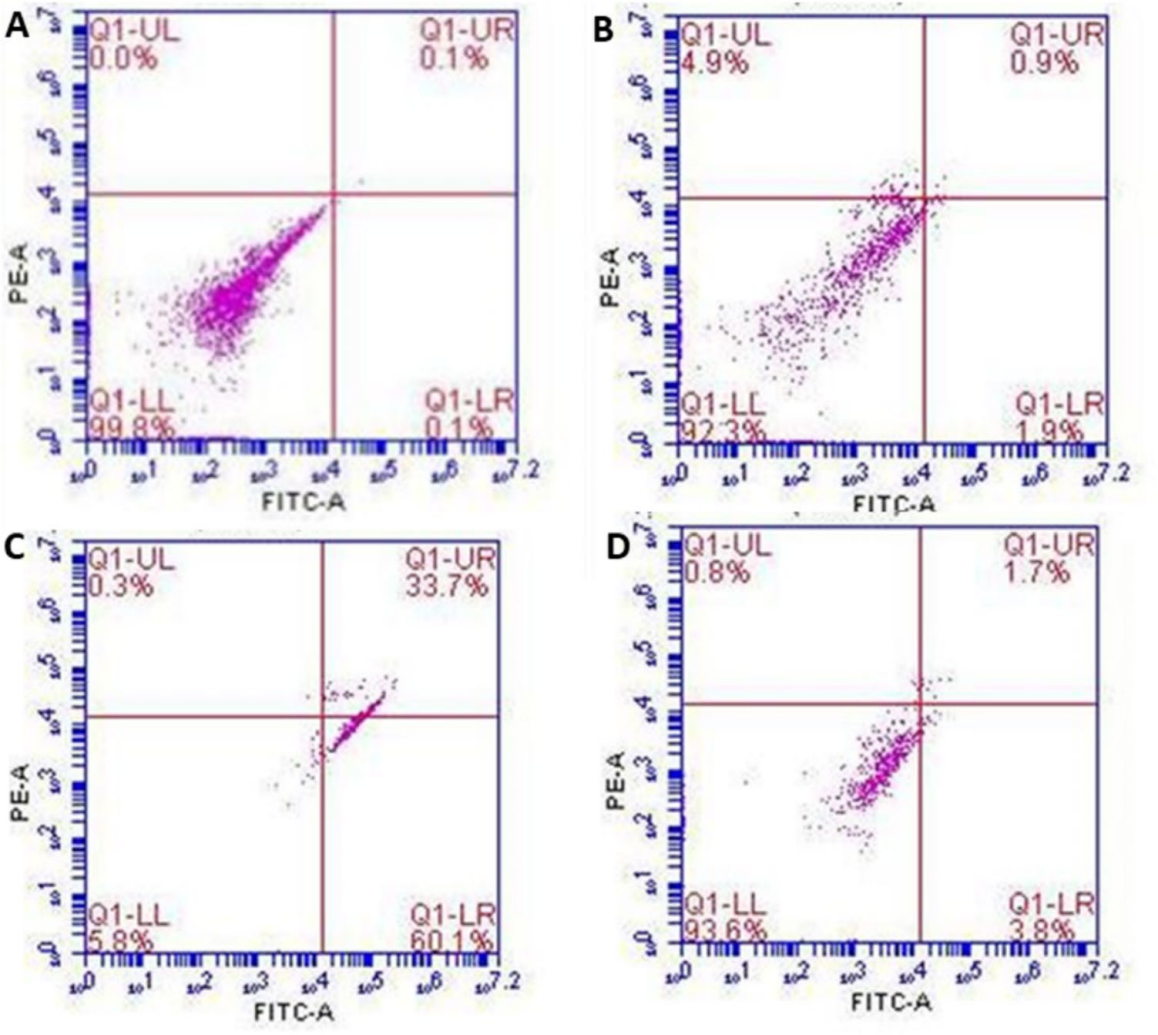
Dot‐plot profile evaluation of cell death by apoptosis using annexin V‐FITC/propidium iodide (PI) staining of BMNCs. Viable cells are represented by the events in the lower left quadrant, and necrotic cells that have absorbed the PI are represented by the events in the upper left quadrant. The lower right quadrant displays the proportion of early apoptotic cells that are simply Annexin V positive, and the top right quadrant displays occurrences illustrating late apoptotic cells that are Annexin V/PI positive. **(A: Control, B: PQS, C: Irradiation & D: Irradiation + PQS group)**

**Fig. 7:**
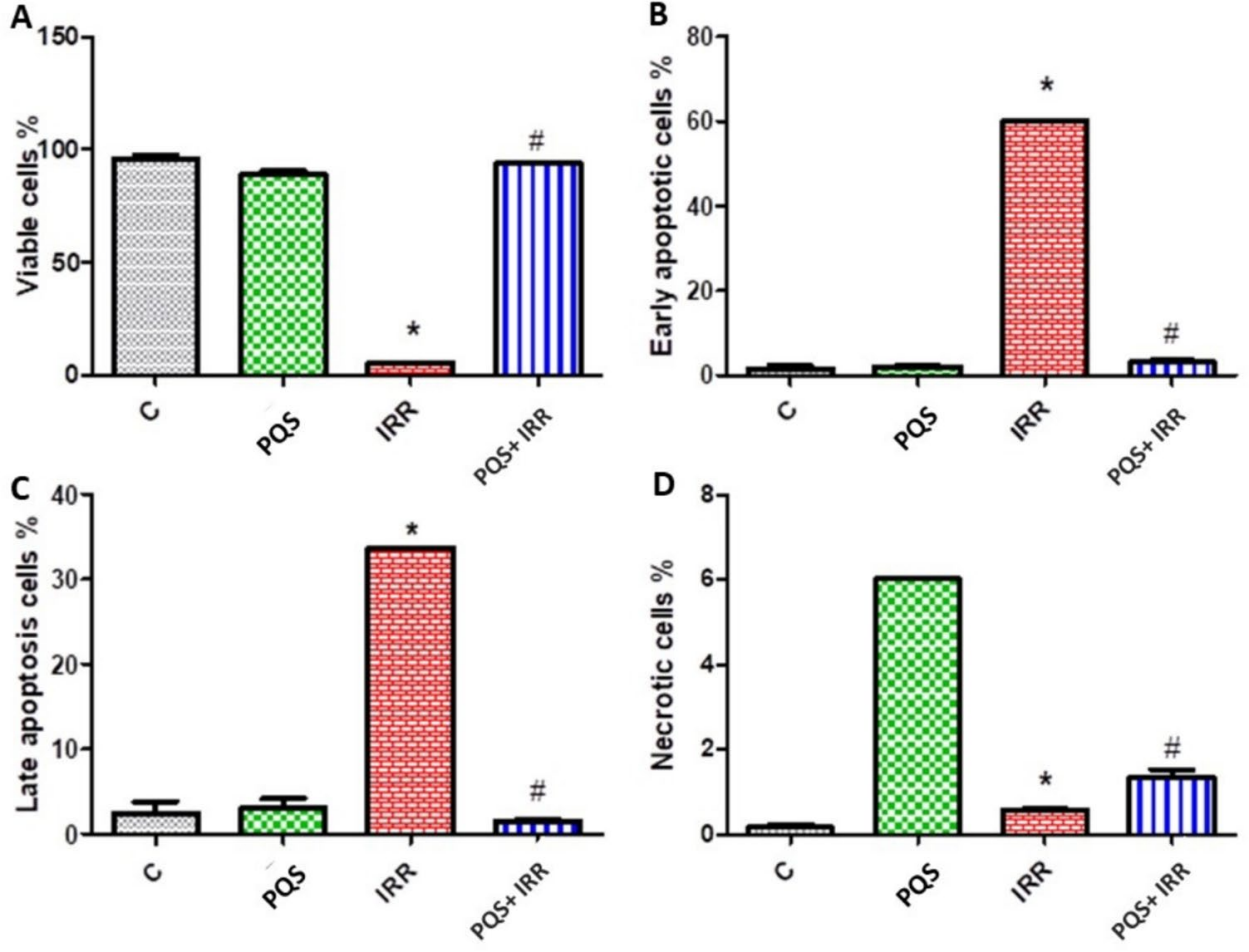
Effect of compound **PQS** on peripheral blood mononuclear cells death in irradiated mice (**A**) Viable cells, (**B**) Early apoptotic cells, (**C**) late apoptotic cells and (**D**) necrotic cells. The results were expressed as mean ± S.D. Statistical analysis was carried out by one-way ANOVA followed by a Bonferroni post hoc comparison test. *: significantly different from control group, #: significantly different from irradiated group at *p* < 0.05. (n = 8)

The percentages of cell viability, necrosis and apoptosis to find out whether **PQS** can prevent radiation‐induced apoptosis were assessed in the peripheral blood mononuclear cells (PBMCs) of various groups using Annexin V‐FITC and PI staining. Analysis of early and late apoptotic cells showed that, gamma irradiation significantly increased early apoptotic (60.1%) and late apoptotic (33.7%) PBMCs compared to the control group (p < 0.05), whereas **PQS** treatment attenuated radiation‐induced damage in irradiated mice by reducing the percentage of early apoptotic to (3.8%) and late apoptotic to (1.7%) (Fig. 6 & 7).

#### Effects of PQS on the histopathological changes in mice kidney injuries induced by gamma radiation

**Fig. 8:**
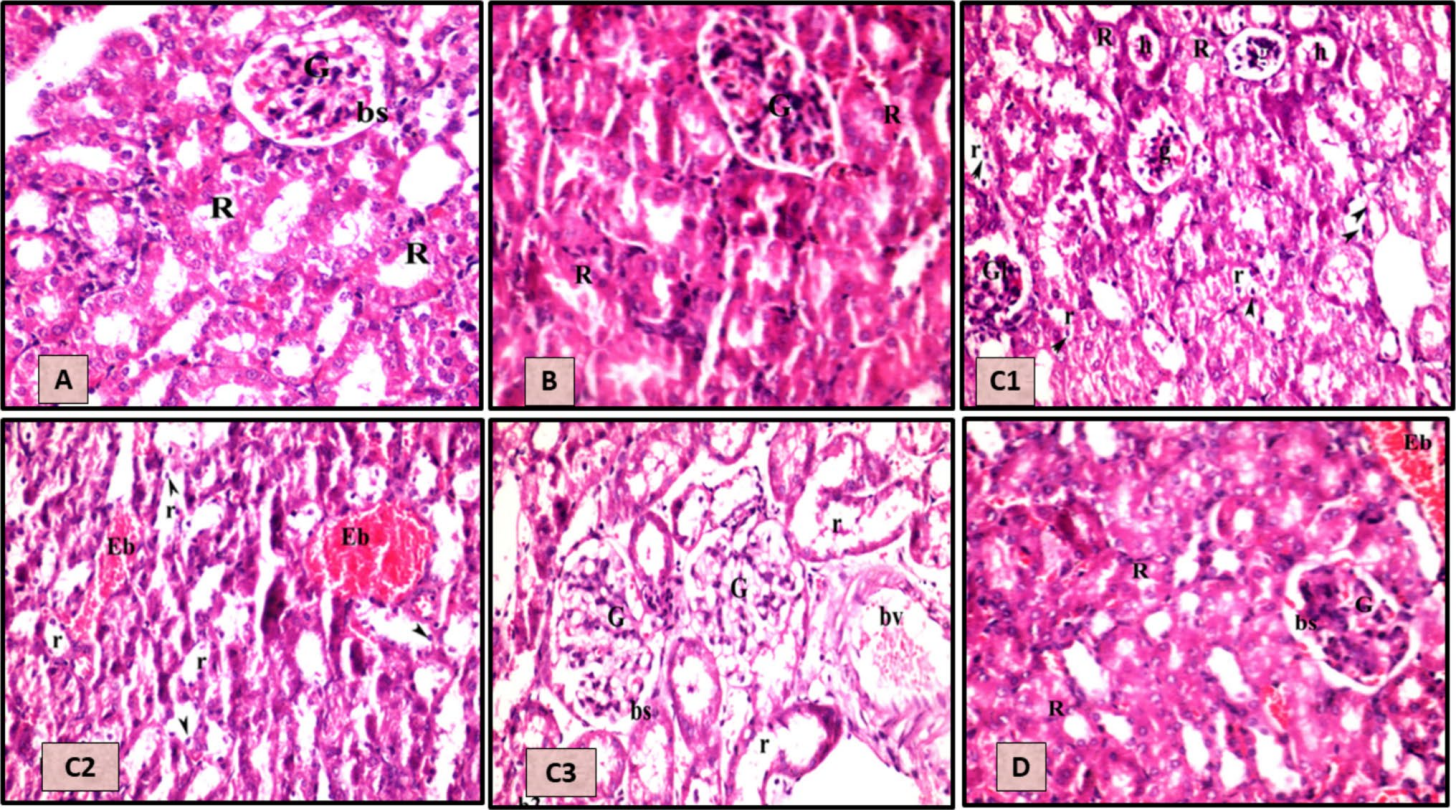
A photograph of a kidney section stained with H&E under light microscope, magnification × 400 (**A**) Control group showed normal appearance of renal tubules (R) and glomerular tuft (G) with bowman's space (bs). (**B**) Drug-treated group showed normal appearance of renal tubules (R) and glomerular tuft (G). **(C1**) Irradiated animals group shows loss of normal architecture, severe cytoplasmic reduction in the epithelial cells lining some renal tubules (r) with a pyknotic nucleus (arrowhead), while others showed cloud swelling (R). Segmented glomerular tuft (G), marked atrophied glomerular tuft (g) and hyline cast in lumen of some renal tubules (h) were observed. (**C2**) Irradiated animals group showed prominent areas of degenerated renal tubules with severe reduction in the cytoplasm of the epithelial cells lining some renal tubules (r) with a pyknotic nucleus (arrowhead), scattered wide areas of extravasated RBCs (Eb). (**C3**) Irradiated animals group showed wide areas of narrow Bowman's space (bs), swelling glomerular tuft (G), degenerated renal tubules with severe reduction of the cytoplasm of the epithelial cells lining some renal tubules (r) with a pyknotic nucleus (arrowhead). Severe dilated blood vessels were also seen (bv). **(D)** Animals from irradiation + **PQS** group showed prominent areas of restored normal appearance of Bowman's space (bs), glomerular tuft (G) and renal tubules (R) with few areas of extravasated RBCs (Eb)

Histological examination of kidney tissues from control group showed normal appearance of renal tubules and glomerular tuft with Bowman's space **(**Fig. 8A**)**. Histological examination of kidney tissues from drug-treated group showed normal appearance of renal tubules and glomerular tuft with Bowman's space **(**Fig. 8B). Histological examination of kidney tissues from irradiated animals showed loss of normal architecture, severe cytoplasmic reduction of the epithelial cells lining some renal tubules with a pyknotic nucleus, while other tubules showed cloud swelling. Segmented glomerular tuft, marked atrophied glomerular tuft and hyaline cast in lumen of some renal tubules (h) were observed (Fig. 8C1). Other regions showed areas of degenerated renal tubules with severe reduction in the cytoplasm of the epithelial cells lining some renal tubules with a pyknotic nucleus, scattered wide areas of extravasated RBCs (Fig. 8C2). Wide areas of narrow Bowman's space, swelling glomerular tuft, degenerated renal tubules with severe cytoplasmic reduction of the epithelial cells lining some renal tubules with a pyknotic nucleus (arrowhead). (Fig. 8C3). Histological examination of kidney tissues from animals subjected to both radiation and drug showed prominent areas of restored normal appearance of Bowman's space, glomerular tuft and renal tubules with few areas of extravasated RBCs **(**Fig. 8D).

#### Effect of PQS on caspase-3, Bax and Bcl2 in kidney of irradiated mice

**Fig. 9:**
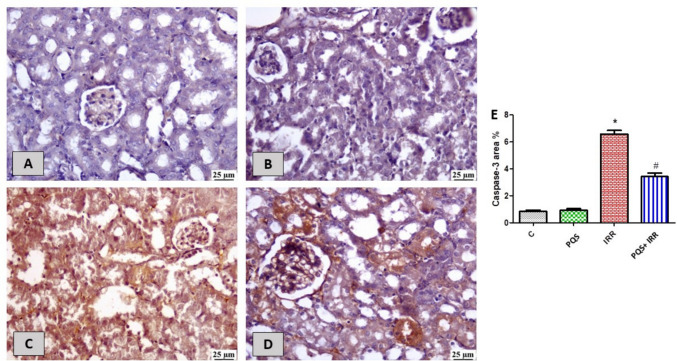
Immunohistostaining showing expression of caspase-3. (**A**) control group showed weak expression. (**B**)** PQS** group showing weak expression. (**C**) Irradiation group showed higher expression. (**D**)** PQS** + irradiation group showing moderate expression (**E**)**.** Effect of compound **PQS** on caspase-3 expression in kidney of irradiated mice. The results were expressed as mean ± S.D. Statistical analysis was carried out by one-way ANOVA followed by a Bonferroni post hoc comparison test. *: significantly different from control group, #: significantly different from irradiated group at p < 0.05. (n = 8)

**Fig. 10:**
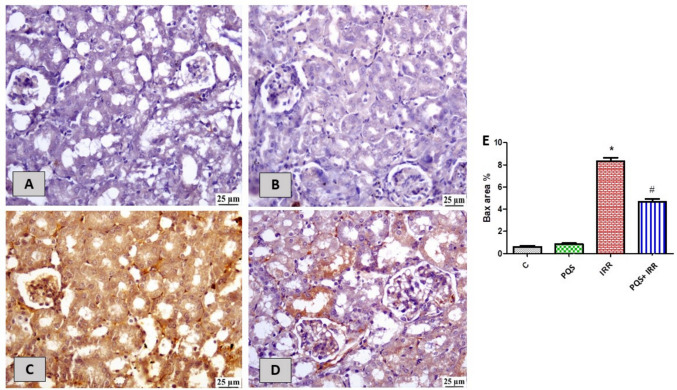
Immunohistostaining showing expression of Bax. (**A**) Control group shows weak expression. (**B**)** PQS** group showing weak expression. (**C**) Irradiation group showed higher expression. (**D**)** PQS** + irradiation group showing moderate expression (**E**)**.** Effect of compound **PQS** on Bax expression in kidney of irradiated mice. The results were expressed as mean ± S.D. Statistical analysis was carried out by one-way ANOVA followed by a Bonferroni post hoc comparison test. *: significantly different from control group, #: significantly different from irradiated group at p < 0.05. (n = 8)

**Fig. 11:**
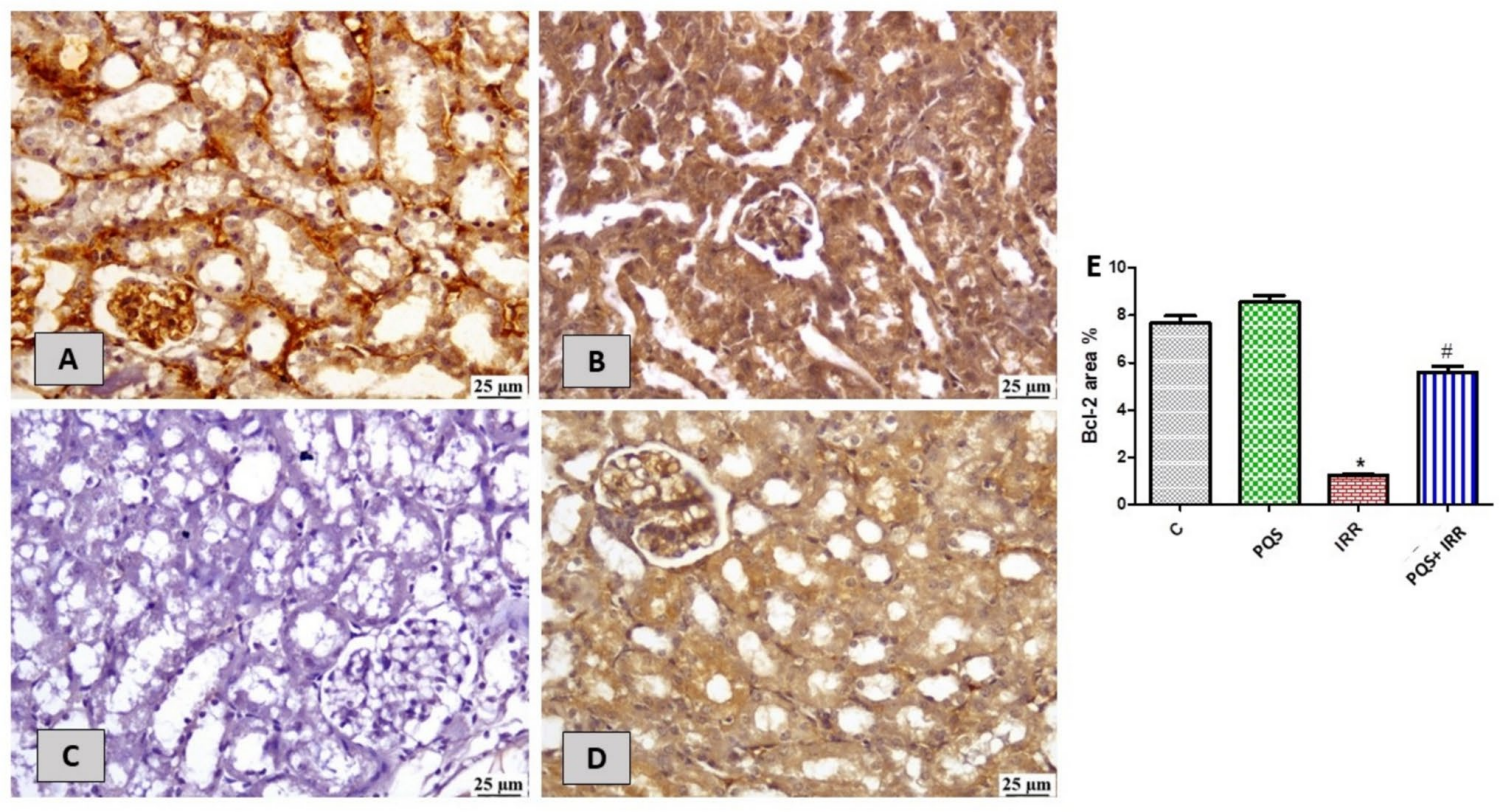
Immunohistostaining showing expression of Bcl2. (**A**) Control group shows high expression. (**B**)** PQS** group showing high expression. (**C**) Irradiation group shows limited expression. (**D**)** PQS** + irradiation group showing moderate expression (**E**) Effect of compound **PQS** on Bcl2 expression in the kidney of irradiated mice. The results were expressed as mean ± S.D. Statistical analysis was carried out by one-way ANOVA followed by a Bonferroni post hoc comparison test. *: significantly different from control group, #: significantly different from irradiated group at p < 0.05. (n = 8)

By assessing caspase-3 using the immunohistochemistry technique, Caspase-3 expression was weak in control groups but significantly elevated in the irradiated (IRR) group compared to the IRR + PQS group **(**Fig. 9**).** Similarly, Bax expression was low in controls but increased in IRR mice relative to all other groups **(**Fig. 10**).** In contrast, Bcl-2 expression was higher in control mice compared to IRR and IRR + PQS groups, indicating reduced anti-apoptotic signaling after irradiation **(**Fig. 11**).**

### *In-silico* ADME study

The success of a compound for therapeutic use depends on factors such as absorption, distribution, metabolism, and excretion (ADME). The bioavailability of drugs is heavily influenced by physicochemical properties. One of the most crucial aspects of drug development is predicting these features before conducting experimental studies. Optimizing the pharmacokinetics of new compounds involves investigating ADME characteristics dynamically. The SwissADME tool (Daina et al. 2017) was used to evaluate the physicochemical properties of the active compound **PQS**. The pharmacokinetic and physicochemical profiles of **PQS** were assessed to predict its drug-likeness and oral bioavailability. As shown in Table 1, **PQS** exhibited a moderate molecular weight (468.51 g/mol) and a total polar surface area (TPSA) of 183.61 Å^2^, which are within the acceptable range for orally active compounds but may slightly limit passive membrane diffusion. The compound demonstrated favorable lipophilicity with a Log*P* of 1.06, indicating balanced hydrophilic–lipophilic properties that could support sufficient solubility and permeability.

**Table 1:** Physicochemical and Pharmacokinetic parameters of **PQS**

Parameters	PQS
TPSA (A^2^)	183.61
n-ROTB	7
Mol. Wt	468.51
Molar Vol	119.48
Log P	1.06
n-HB donor	2
n-HB acceptor	8
Lipinski’s violation	0
GI absorption	Low
CYP1A2 inhibitor	No
CYP3A4 inhibitor	Yes
Log *K*_p_ (skin permeation)	−8.42 cm/s
Bioavailability score	0.55
PAINS	0 alert
Synthetic accessibility	3.27

**PQS** complied fully with Lipinski’s rule of five, showing no violations, which supports its potential as a drug-like molecule. The presence of two hydrogen bond donors (HBD) and eight acceptors (HBA), along with seven rotatable bonds (ROTB), suggests moderate molecular flexibility that is conducive to target binding. Despite its compliance with drug-likeness rules, **PQS** showed low gastrointestinal (GI) absorption, likely attributed to its relatively large polar surface area and molecular size.

Regarding metabolic stability, **PQS** was predicted to be a CYP1A2 non-inhibitor but a CYP3A4 inhibitor, suggesting possible metabolic interactions at the level of cytochrome P450 isoenzymes. Its skin permeation (Log K_p_ = –8.42 cm/s) indicated poor transdermal diffusion, while a bioavailability score of 0.55 reflects moderate systemic exposure upon oral administration. Furthermore, the compound was devoid of PAINS (pan-assay interference) alerts, highlighting a low probability of false-positive bioactivity, and exhibited a synthetic accessibility score of 3.27, implying feasible laboratory synthesis. Collectively, these parameters demonstrate that **PQS** possesses a balanced physicochemical profile with drug-like properties.

## Discussion

Ionizing radiation (IR) therapy is a cornerstone in the treatment of cancer; nevertheless, its potential is often accompanied by damage in the healthy tissues, a phenomenon called radiation-induced normal cell toxicity (De Ruysscher et al. 2019; Kim et al. 2014). Significant concerns had evolved from radiation-induced kidney damage, as it is a highly vascularized organ with complex cellular architecture, leading to radiation nephropathy (Klaus et al. 2021). This damage usually involves apoptosis (programmed cell death) pathways in the renal cells (Jiao et al. 2022). Consequently, it is crucial to find new anti-cancer compounds that can protect renal tissues from radiotherapy oxidative and apoptotic effects, to improve the therapeutic window of radiotherapy.

The pyrazine-linked quinazolinone sulfonamide derivative (**PQS**) was found to have a wide range of biological activity, including radioprotective and anticancer effects (Deng et al. 2025; Soliman et al. 2021). The present research has begun to check the safety margin of **PQS** on Vero normal cell line, and compare its cytotoxic effect with that of ascorbic acid (standard antioxidant drug). Furthermore, *in vivo* impact of counteracting irradiation-induced cellular damage, focusing on oxidative stress and apoptosis in renal tissues, was examined.

The present results revealed a significant increase in the levels of ROS, MDA and NO in kidney tissues of the irradiated mice compared with control group. While the level of GSH and the activity of GST enzyme were decreased. It was accompanied by a significant increase in the levels of urea and creatinine, reflecting an impairment of kidney function. This goes in agreement with other studies that found that gamma irradiation induces disequilibrium between antioxidant and oxidative stress in renal tissue, in addition to impairment of kidney function (Abdel-Magied and Elkady 2019; Özyurt et al. 2014; Saif-Elnasr et al. 2023). The damage induced by IR is primarily mediated by ROS, leading to a cascade of events including DNA damage, oxidative stress, inflammation and apoptosis (Wilkins et al. 2019). In addition to the depletion of antioxidant enzymes exhausted in counteracting the effect of oxidative stress (Mekkawy et al. 2020; Ozougwu 2016).

Severe, persistent DNA damage and oxidative stress trigger apoptosis along with impairments in renal endothelial, tubular and glomerular cells (Aziz et al. 2018; Soliman et al. 2019a), as appeared in histopathological examination. Moreover, the detection of apoptosis in PBMCs showed an increase in the early and late apoptotic cells in the irradiated groups. Furthermore, the immuno-histochemical assessment of the apoptotic pathway revealed a significant increase in the expression of caspase-3 and Bax, with a significant decrease in Bcl2 expression in the IRR group compared to the control. Radiation induces apoptosis in renal cells, contributing to organ dysfunction, probably due to oxidative stress and prolonged DNA damage, which in response activates p53 (Changizi et al. 2021; Manna et al. 2015; Mukherjee et al. 2022). Activated p53 then acts as a transcription factor regulating cell cycle arrest and apoptosis (Chen 2016).

On the other hand, **PQS** exhibited a promising effect against IR-induced kidney damage by decreasing serum urea and creatinine levels, as well as ROS, MDA and NO, while increasing the antioxidant levels (GSH and GST) in kidney tissues. In addition, **PQS** succeeded in protecting renal cells from apoptosis by upregulating the early and late apoptosis in PBMCs, in addition to decreasing the expression of caspase-3, Bax and ameliorating the expression of Bcl2 in renal cells. This impact may be caused by several mechanisms. Firstly, through antioxidant activity, many quinazolinone derivatives possess direct free radical scavenging power, enabling them to neutralize ROS before causing DNA and cellular damage. This can be endorsed by their chemical structure, which usually allows for electron donation and free radical stabilization (Hricovíniová et al. 2021; Soliman et al. 2020a). Secondly, activation of the antioxidant defense mechanisms, some quinazolinone derivatives were observed to activate nuclear factor erythroid 2-related factor 2 (Nrf2) pathway (Soliman et al. 2020b, 2021). Nrf2 is the key cellular defense regulator against oxidative stress, promoting the activation of the antioxidant enzymes like NQO1, SOD and GST, as well as increasing GSH levels (Holmström et al. 2016). Finally, the anti-apoptotic effect, by modulating key proteins involved in the apoptosis pathways. This could involve inhibition of the pro-apoptotic factors (Bax and caspase-3), or upregulating the anti-apoptotic markers like Bcl-2, or stabilizing the mitochondrial membrane by preventing cytochrome C release from mitochondria (Gogvadze et al. 2006; Mehndiratta et al. 2016; O'Brien and Kirby 2008).

This study was extended to explore the anticancer effect of **PQS** against HepG-2 and HeLa cell lines, comparing its cytotoxic impact with that of doxorubicin, a well-established chemotherapeutic agent. The IC_50_ of **PQS** was as potent as doxorubicin on HeLa cells and less potent using HepG-2 cells, but still showed a promising cytotoxic effect. So, it suggests that it holds a potential anticancer effect. The pyrazine-linked quinazolinone sulfonamide derivative contains quinazolinone, which is a kinase inhibitor and could interact with DNA/topoisomerase. In addition to the sulfonamide moiety that improves the binding with carbonic anhydrase causes its inhibition. Also, it contains pyrazine, which is an electron-deficient heterocycle enhancing receptor binding and ROS modulation, and when linked together, gives a synergistic anticancer activity that may account for the possibility of **PQS** as a promising anticancer compound (Alqahtani et al. 2022; Deng et al.^2025^).

## Conclusion

**PQS** demonstrates a notable cytotoxic effect on HepG-2 and HeLa cells, indicating its potential as an anticancer agent. Additionally, it can significantly boost the kidney’s ability to combat IR-induced oxidative and apoptotic insult. Despite the fact that studies focusing on the radioprotective and the anti-apoptotic effect of **PQS** or any quinazolinone derivatives on the kidney are still emerging, previous research on their radioprotective effect in liver provides promising insights. For instance, novel iodinated quinazolinones bearing sulfonamide have shown a reduction in radiation-induced liver damage, evidenced by lowering MDA and ROS, increasing the antioxidant defense pathways and lowering mortality in mice, as previously stated. Altogether, given the commonality of oxidative stress and apoptosis as major radiation injury mechanisms, the present findings strongly suggest **PQS** as a novel radio protector for a similar impact on the kidney tissues.

Further studies are needed to specifically investigate the effect of **PQS** on the radiation-induced nephropathy in different renal cell types and long-term kidney function. As well as elucidating the molecular mechanisms targeting signal pathways of **PQS** as an anticancer agent, and testing **PQS** against Renal Carcinoma Cell Line (RCC). Also, the study of the pharmacokinetic profile for kidney-specific protection and potential side effects and comparing the effect of **PQS** to a well-established radioprotector would be an added value.

## Data Availability

All source data for this work (or generated in this study) are available upon reasonable request.
